# Tau fibril with membrane lipids: Insight from computational modeling and simulations

**DOI:** 10.1371/journal.pone.0258692

**Published:** 2021-10-15

**Authors:** Prechiel A. Barredo, Marvin Jose F. Fernandez, Christopher E. Ambe, Mannix P. Balanay

**Affiliations:** 1 Department of Chemistry, Iligan Institute of Technology, Mindanao State University, Iligan, Republic of the Philippines; 2 Department of Chemistry, Nazarbayev University, Nur-Sultan, Kazakhstan; Beijing Foreign Studies University, CHINA

## Abstract

The microtubule-binding protein tau has been the center of researches concerning Alzheimer’s disease (AD) due to several clinical trials of β-amyloid therapies failing recently. The availability of the tau fibril structure from AD brain enables computational modeling studies to calculate binding affinities with different ligands. In this study, the tau paired helical filaments (PHF-Tau) (PDB ID: 5O3L) was used as receptor and interactions with the lipids: 3-alpha-cholesterol; 1-palmitoyl-2-oleoyl-sn-glycero-3-phosphocholine; and C18:1 sphingomyelin, were explored with molecular docking, molecular dynamics, and natural bond orbital analysis. Docking sites upon solvation of the protein with transferable interatomic potential-3 points reveal the amphipathic nature of PHF-Tau and molecular dynamics simulations show that the embedded phosphocholine at the tail side gives high potential energy values with some amino acids forming H-bond interactions.

## 1. Introduction

Alzheimer’s disease (AD) is the most common form of dementia, which was discovered in 1906 that affects the aged population that until now there are no mechanism-based therapies [1]. The major characteristic of AD is the decline in cognitive functions of elderly individuals, such as memory, speech, and reasoning [2,3]. As of to date, there are four drugs that have been approved and currently used in symptomatic treatment for AD [4]. Three of them are classified as cholinesterase inhibitors namely: donepezil [5], rivastigmine [6], and galantamine [7], and the other approved drug is based on N-methyl-D-aspartate receptor antagonist which is the memantine [8]. In June 2021, the US Food and Drug Administration (FDA) has approved Aducanumab as AD treatment which is the first of its kind to treat the cause of the disease rather than the symptom [9]. Aducanumab is an intravenously infused antibody that targets amyloid-β (Aβ) plaque deposits [10]. However, the approval by US FDA sparked some concern from the scientific community since there is not enough evidence to show that the treatment can slow cognitive decline [9].

Basically there are two main pathological hallmarks for AD, these are the neuritic plaque deposits of Aβ peptide and the neurofibrillary tangles made up of tau filaments from microtuble-binding protein tau [11,12]. The tau filaments were shown to be formed from full-length tau in cells [13–15] and a gradual deposition of abnormal tau in neurons occurs in the form of either neurofibrillary tangles (NFTs) or neuronal threads (NT) wherein the AD pathology follows strictly defined stages [16,17]. It was observed in the previous studies that the correlation between β-amyloid plaques and the cognitive decline in the symptomatic phase of dementia were weak or even non-existent [18–20] that prompted various researchers to moved their focus from Aβ to tau [21–23]. Tau protein is a microtubule-binding protein whose primary function is in the assembly and stabilization of microtubules. Phosphorylation and *O*-glycosylation are the most common post translational modification of tau proteins. Phosphorylation changes tau protein structure and regulates its biological activity [24–26]. Tau pathology propagation could be promoted by *O-*glycosylation or acetylation, which refers to the addition of *O*-linked N-acetylglucosamine (*O*-GlcNAc) on Ser or Thr residues near Pro residues [27–29].

**Fig 1:**
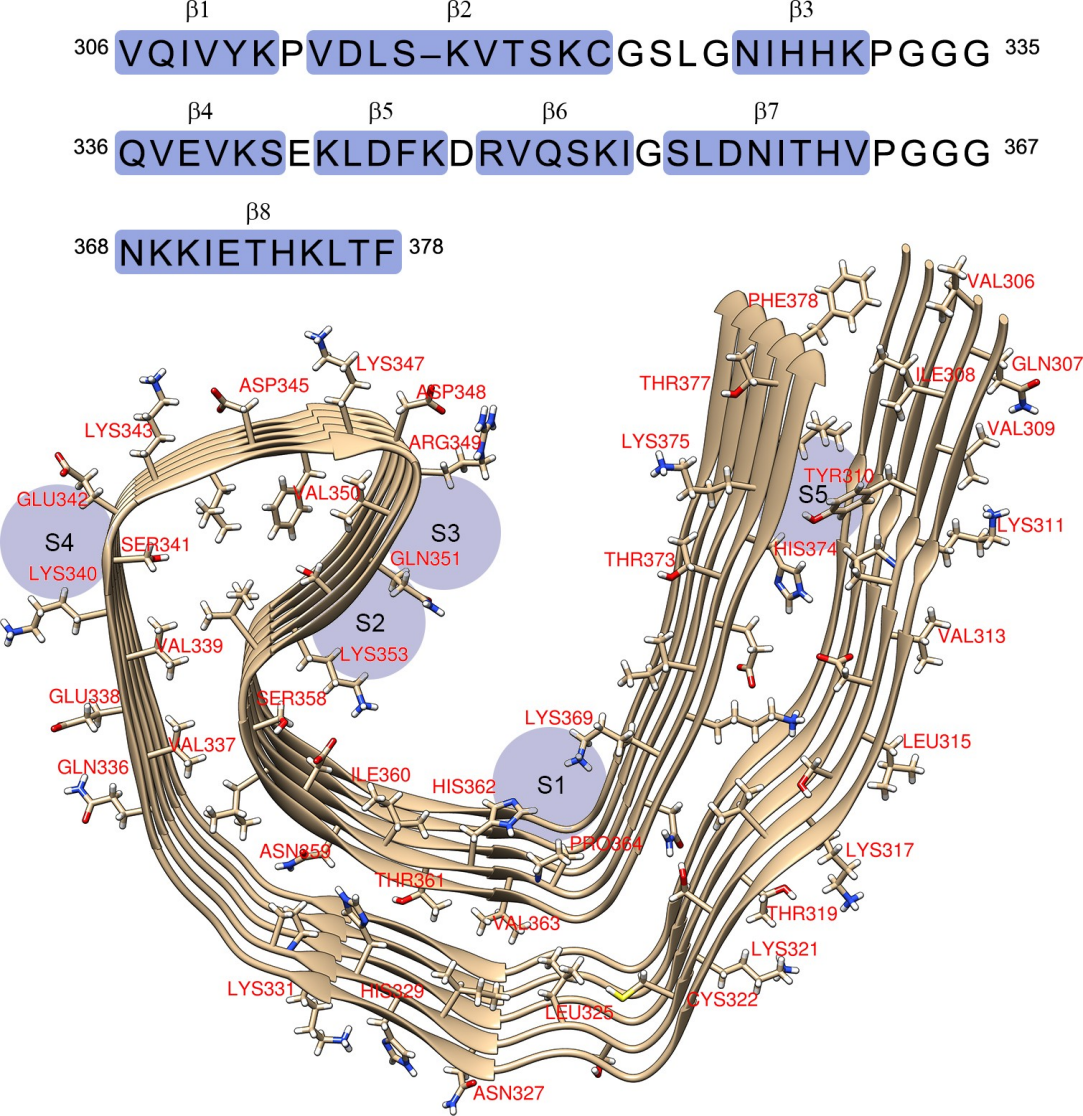
Amino acid sequence of the PHF-Tau protein with the observed b-strand regions and the corresponding schematic view of the C-shaped architecture of the protofilament core with possible high-affinity binding sites of the protofibril (S1 to S5) [35,36].

Alois Alzheimer, who was credited to the identification of the disease known as presenile dementia later renamed as AD after him, indicated that there was a third, often ignored, pathological hallmark of AD, wherein an AD brain displays a higher occurrence of “adipose inclusions” or “lipoid granules” which suggests abnormal lipid metabolism [30]. Researchers found that perturbed sphingomyelin (SM) and/or phospholipid metabolites are one of the main contributors to the neuron degeneration and dysfunction that are commonly found in AD [31]. The lipids, C18:0 spingomyelin (d18:1/18:0) (*N*-octadecanoyl-sphing-4-enine-1-phosphocholine) and its derivative C18:1 spingomyelin (d18:1/18:1(9Z)) (*N*-(9*Z*-octadecenolyl)-sphing-4-enine-1-phosphocholine) have been used in the search of additional biomarkers for AD, where they were found to be considerably higher in cerebrospinal fluids (CSF) of patients displaying pathological levels of amyloid-β_42_, total tau, and phosphorylated-tau-181 [32]. The 1-palmitoyl-2-oleoyl-sn-glycero-3-phosphocholine (also known as 1-POPC or PC(16:0/18:1(9Z)) is one of the most common one-component system to study the cell membrane properties. It was chosen since the phosphatidylcholine (PC) is the most abundant headgroup in mitochondrial-like phospholipid membrane and the 16:0/18:1 chain composition is common in naturally-extracted PC [33]. It was shown that phospholipids can form highly stable interaction with paired helical filaments (PHF-Tau) through the C-terminal fragment containing four microtubule-binding repeats of Tau [34]. The structure of PHF-Tau in AD consists of an ordered core of a pair of protofilament with C-shaped architecture consisting of amino acid residues 306–378 based on cryo-electron microscopy for an AD brain as receptor (PDB ID:5O3L) is shown in Fig 1 [15,35].

**Fig 2:**
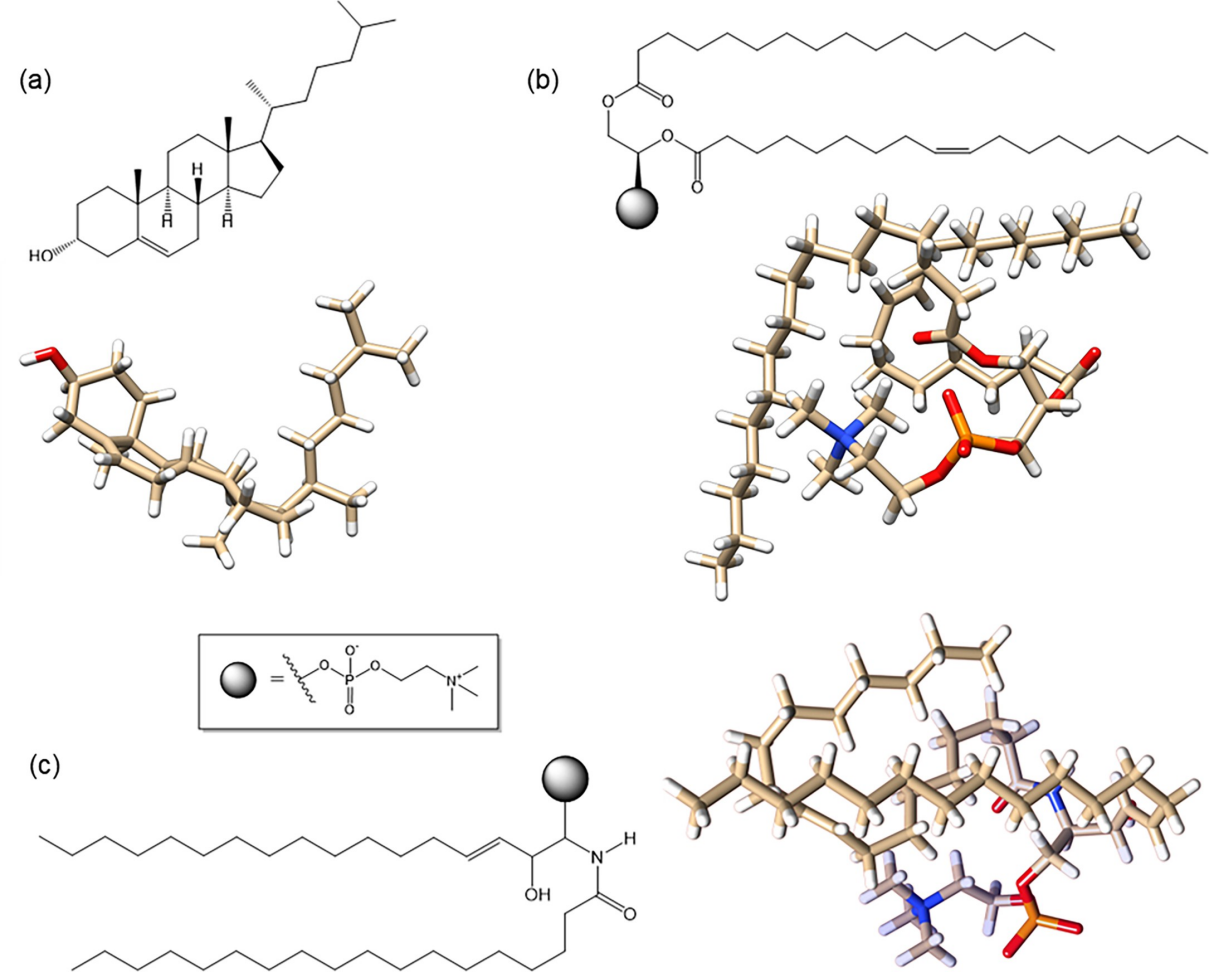
Molecular structures and its 3D geometric representations of (a) 3-alpha-cholesterol, (b) 1-palmitoyl-2-oleoyl-sn-glycero-3-phosphocholine, and (c) C18:1 sphingomyelin.

In this study, a theoretical modelling approach using 3D structure of the Tau and representative lipids as ligands such as 3-alpha-cholesterol (cholesterol), 1-palmitoyl-2-oleoyl-sn-glycero-3- phosphocholine (1-POPC), and C18:1 sphingomyelin (Fig 2) is reported using molecular docking to investigate the ligand binding sites in PHF-Tau. Molecular dynamics simulation is used to show the movement and stability of the complexes obtained, and natural bond orbital (NBO) analysis allows further interpretation of the strength of bonding between the ligands and the protein obtained during docking. This paper aims to give computationally determined structures of PHF-Tau—membrane lipid complexes and to study the interactions involved which could lead to discovery of other compounds that have effect on the protein as well and ultimately develop new drugs against AD.

## 2. Computational details

### 2.1. Molecular docking

All calculations were based on the 3D structure of PHF-Tau (PDB ID:5O3L) obtained using cryo-EM method with a resolution of 3.4 Å downloaded from the protein data bank [35]. The UCSF Chimera software version 1.13.1 [37] was used to visualize and prepare the protein of interest for docking of ligands. Incomplete side chains of the protein were replaced using the Dunbrack rotamer library [38] before molecular docking calculations with AutoDock Vina [39]. The standard residues were assigned with parameters from the Amber ff14SB force field [40]. The unbound ligands were obtained from PubChem database using their simplified molecular-input line-entry system (SMILES) string and then minimized with AM1-BCC (a semi-empirical AM1 with bond charge correction (BCC)) parametrized to reproduce *ab initio* (HF/6-31G(d)) electrostatic potentials [41]. Docked conformations with excellent to good root-mean-square deviations (RMSD) were then searched for hydrogen bonds to see protein-ligand interactions. In order to assess the correctness of the docked structure, the difference between the minimized and the bound structure of an identical molecule should have an RMSD of less than 1.5 Å. The cut-off RMSD of 2 Å is usually set as a good criterion of the correct bound structure prediction [39].

### 2.2 Analysis of docked conformations

#### 2.2.1 Molecular dynamics

Docked conformations of the ligands were analyzed using molecular dynamics simulation provided by Molecular Modeling Toolkit (MMTK) which was included in the Chimera software [37]. An equilibration protocol consisting of 50000 steps (timestep = 1 fs) at a temperature gradient from 0 to 298 K at 10 K/ps using Nose thermostat was utilized. Finally, the system was subjected to production MD run at 298 K with a relaxation time of 0.2 ps. Amber ff14SB force field (40) was used for standard residues and for the nonstandard residues Amber’s Antechamber module was utilized. Plots of potential energies, kinetic energies and temperature were obtained for 50000 steps of equilibration and production dynamic phase equal to 50 ps simulation time.

#### 2.2.2 Natural Bond Orbital (NBO) analysis

Natural bond orbital calculations were performed using NBO 3.1 program [42] as implemented in the Gaussian 16 [43] package with B3LYP [44,45] hybrid exchange-correlation functional and 6-31G(d) basis set. The NBO analysis calculates intermolecular delocalization or hyperconjugation through second-order perturbation theory. Filled-orbitals (donor) of one system and vacant-orbitals (acceptor) of another system are the ones that interact. For each donor NBO (*i*) and acceptor NBO (*j)*, the stabilization energy or the second-order interaction energies, $\Delta E_{ij}^{\left(2\right)}$, was calculated by:

$$\Delta E_{ij}^{\left(2\right)}=q_{i}\frac{F{\left(i,j\right)}^{2}}{\varepsilon_{j}-\varepsilon_{i}}$$ (1)

where *q*_*i*_ is the donor orbital occupancy, *ε*_*i*_ and *ε*_*j*_ are diagonal elements (orbital energies) and *F*(*i*,*j*) is the off-diagonal NBO Fock matrix element [46].

### 2.3 Docking to solvated protein

A solvated PHF-Tau protofilament was prepared in the Visual Molecular Dynamics (VMD) software version 1.9.3 [47] using the 3-site TIP3P (transferable intermolecular potential with 3 points) water model before it was docked with the optimized ligands. The ligands were optimized with AM1-BCC and with B3LYP/6-31G(d) theoretical methodologies.

## 3. Results and discussion

### 3.1 Molecular docking and NBO analysis

The molecular docking studies were performed for 3-alpha-cholesterol, 1-POPC and C18:1 sphingomyelin at the binding site of the PHF-Tau to get an insight on the intermolecular interactions between the protein and the ligand. Before docking, the protein was prepared using dock prep in Chimera software [37] and the ligands were optimized using AM1-BCC [41]. After docking, all the stack of docking poses was visually checked and multiple stack conformations were selected based on their docking energies and RMSD values. Finally, the best conformation for each ligand were finally chosen, and their binding energies and RMSD were calculated.

#### 3.1.1 3-alpha-cholesterol

**Fig 3:**
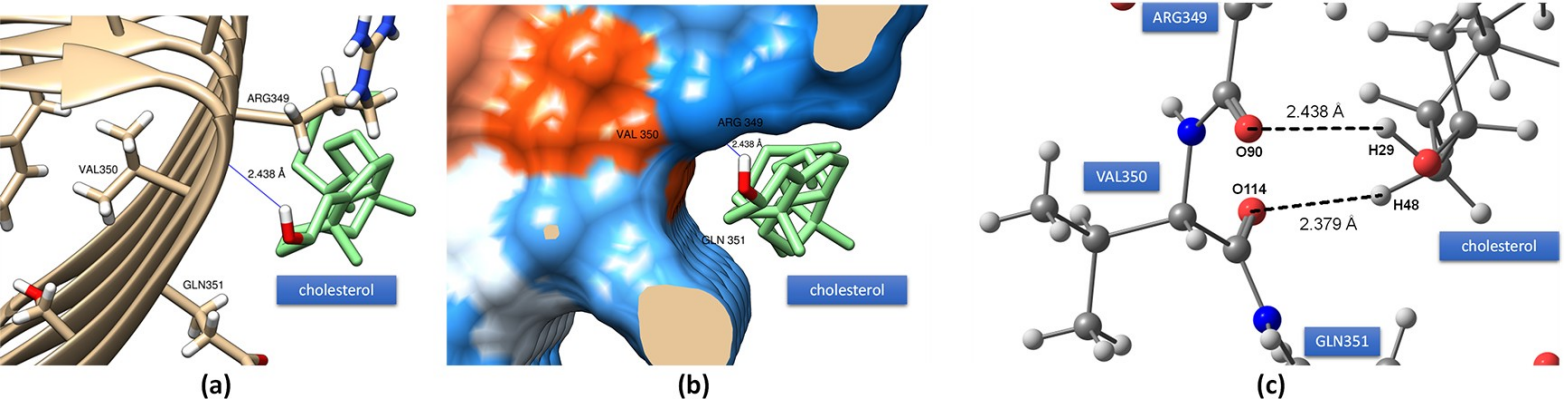
(a) The docking conformation of 3-alpha-cholesterol on PHF-Tau showing the H-bond formation between the PHF-Tau backbone and OH group of the ligand. (b) Electrostatic surface potential model on the docking site of 3-alpha-cholesterol on PHF-Tau which shows the hydrophobicity of the binding site (blue regions are hydrophilic and red orange regions are hydrophobic). (c) H-bonds obtained in NBO analysis.

Fig 3 shows the docking of 3-alpha-cholesterol on PHF-Tau protein which shows the same docking site (site S3, Fig 1) is obtained for some of the Tau tracers on PHF-Tau [36]. Due to the size of the cholesterol, it could easily fit the small crevice between ARG349 and GLN351. The H-bond formation was from the hydroxyl moiety of the cholesterol with the protein backbone of the PHF-Tau protofilament with a binding affinity of -7.5 kcal mol^-1^ (Fig 3A). This type of bonding was qualitatively viewed in the surface map of the protein wherein the hydrophilic part of the protein forms the binding site of the 3-alpha-cholesterol (Fig 3B). To further analyze this bonding, a natural bond orbital calculation using B3LYP/6-31G(d) theoretical methodology was applied. As shown in Fig 3C, an intermolecular H-bond was formed between the O90 from the carboxylic end of ARG349 and H29 of the hydroxyl group of 3-alpha-cholesterol with a stabilization energy of 1.64 kcal mol^-1^. Another bond where the oxygen atom of the amide moiety of VAL350 is the donor NBO and the hydrogen attached to carbon nearest it as acceptor NBO with stabilization energy of 1.57 kcal mol^-1^ was obtained. This bidentate-type of binding makes it very stable conformation between the protein and the ligand. The interatomic bond length was found to be at 2.438 Å and 2.379 Å for π(C89-O90) → σ*(O28-H29) and π(C113-O114) → σ*(C15-H48), respectively.

#### 3.1.2 1-Palmitoyl-2-Oleoyl-sn-Glycero-3-Phosphocholine

**Fig 4:**
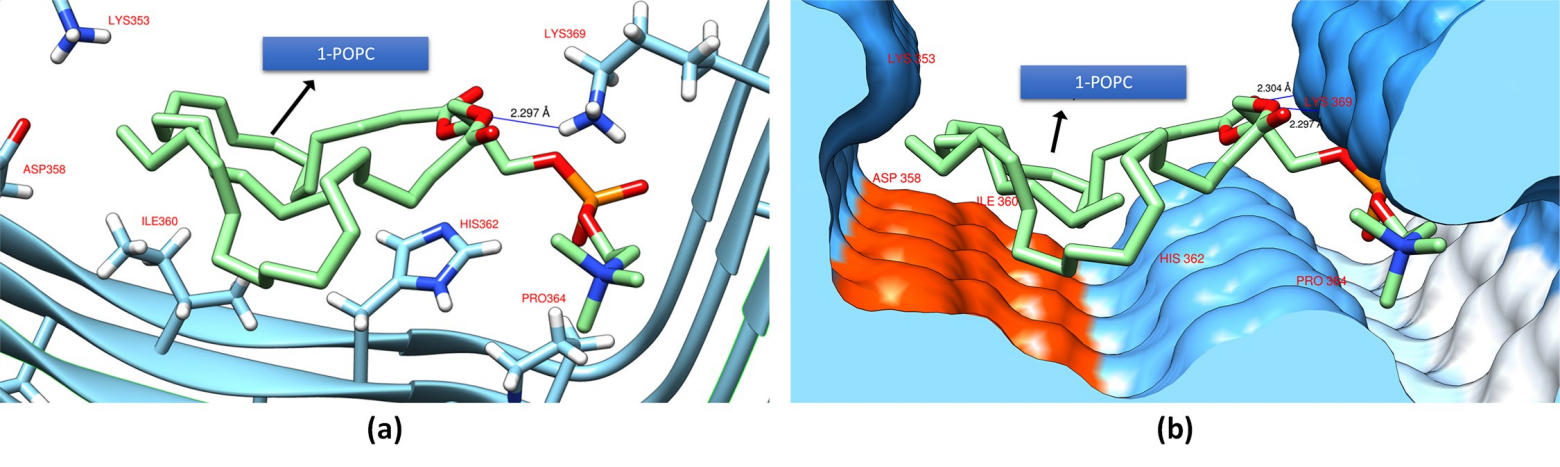
(a) The docking conformation of 1-POPC on PHF-Tau showing the H-bond formation between 1-POPC and the PHF-Tau backbone. (b) Electrostatic surface potential model on the docking site of 1-POPC ON PHF-Tau which shows the hydrophobicity of the binding site (blue regions are hydrophilic and red orange regions are hydrophobic).

**Fig 5:**
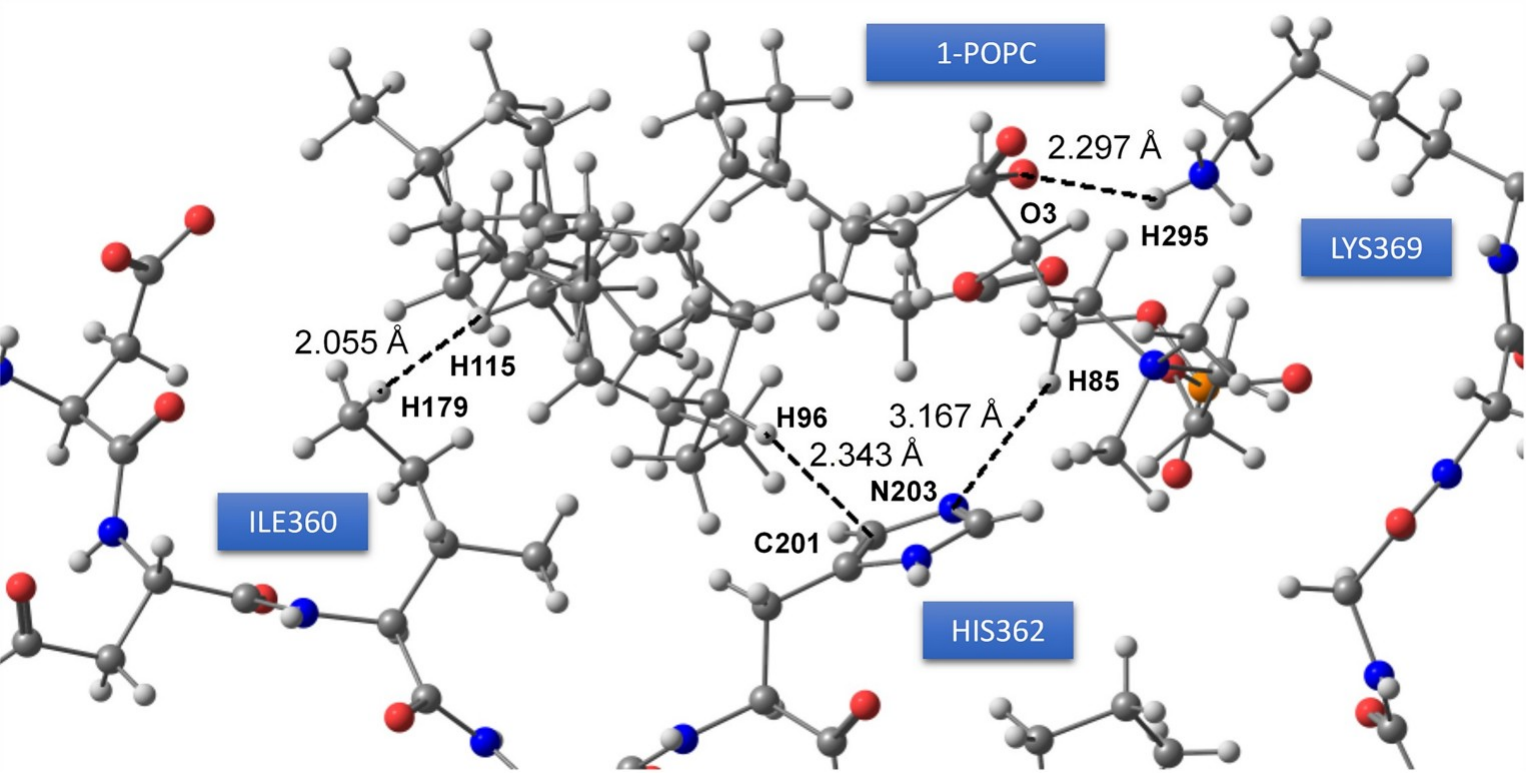
Intermolecular interactions between 1-POPC and PHF-Tau based on NBO analysis. Interatomic distance is shown in angstrom.

Fig 4 shows the docking of 1-POPC on PHF-Tau protein. Unlike the cholesterol, 1-POPC was docked at the S1 binding site (Fig 1) which interacts with LYS369, HIS362, and ILE360 residues with a binding affinity of -5.2 kcal mol^-1^. The hydrophilic part of the protein as shown in the hydrophobicity surface map (Fig 5B) located at the middle area with a deep groove forms the hydrogen bond with an interatomic distance of 2.297 Å. NBO analysis was done to properly look into the contributing intermolecular interactions between the protein and the ligand. As shown in Fig 5, there are four significant contribution with a stabilization energies of 2.04, 1.39, 0.98, and 0.74 kcal mol^-1^ for π(C199-C201) → σ*(C39-H96), LP (O3) → σ*(N284-H295), σ(C168-H179) → σ*(C49-H115), and π(C202-N203) → σ*(C21-H85), respectively. Interestingly, stronger stabilization energy was observed for the London dispersion forces π(C199-C201) → σ*(C39-H96) as compared to the hydrogen bond formation between the glycerol of 1-POPC and amine side group of LYS369 (LP (O3) → σ*(N284-H295)). Further analysis revealed that the contributing oxygen was from the C-O-C moiety rather than coming from the oxygen of the carbonyl group. The higher stabilization energy of 2.04 kcal mol^-1^ found between the imidazole side chain of HIS362 and the fatty acid moiety of 1-POPC (π(C199-C201) → σ*(C39-H96)) was probably due to the resonance effect.

#### 3.1.3 C18:1 sphingomyelin

**Fig 6:**
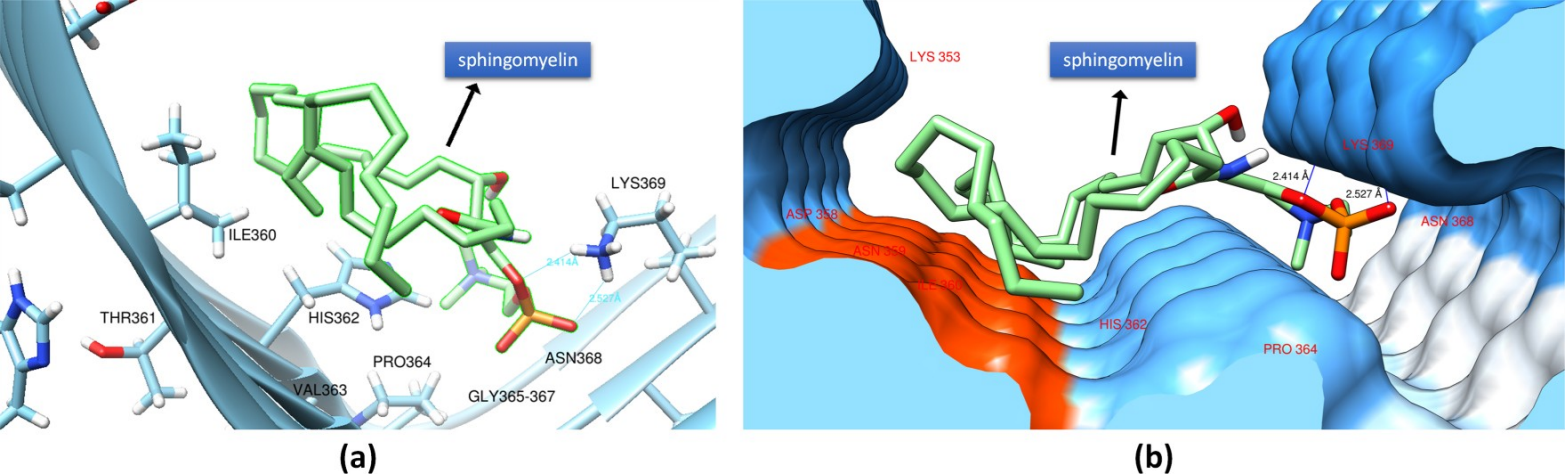
(a) The docking conformation of C18:1 Sphingomyelin on PHF-Tau showing the H-bond formation between C18:1 Sphingomyelin and the PHF-Tau backbone. (b) Hydrophobicity of the binding site (blue regions are hydrophilic and red orange regions are hydrophobic).

**Fig 7:**
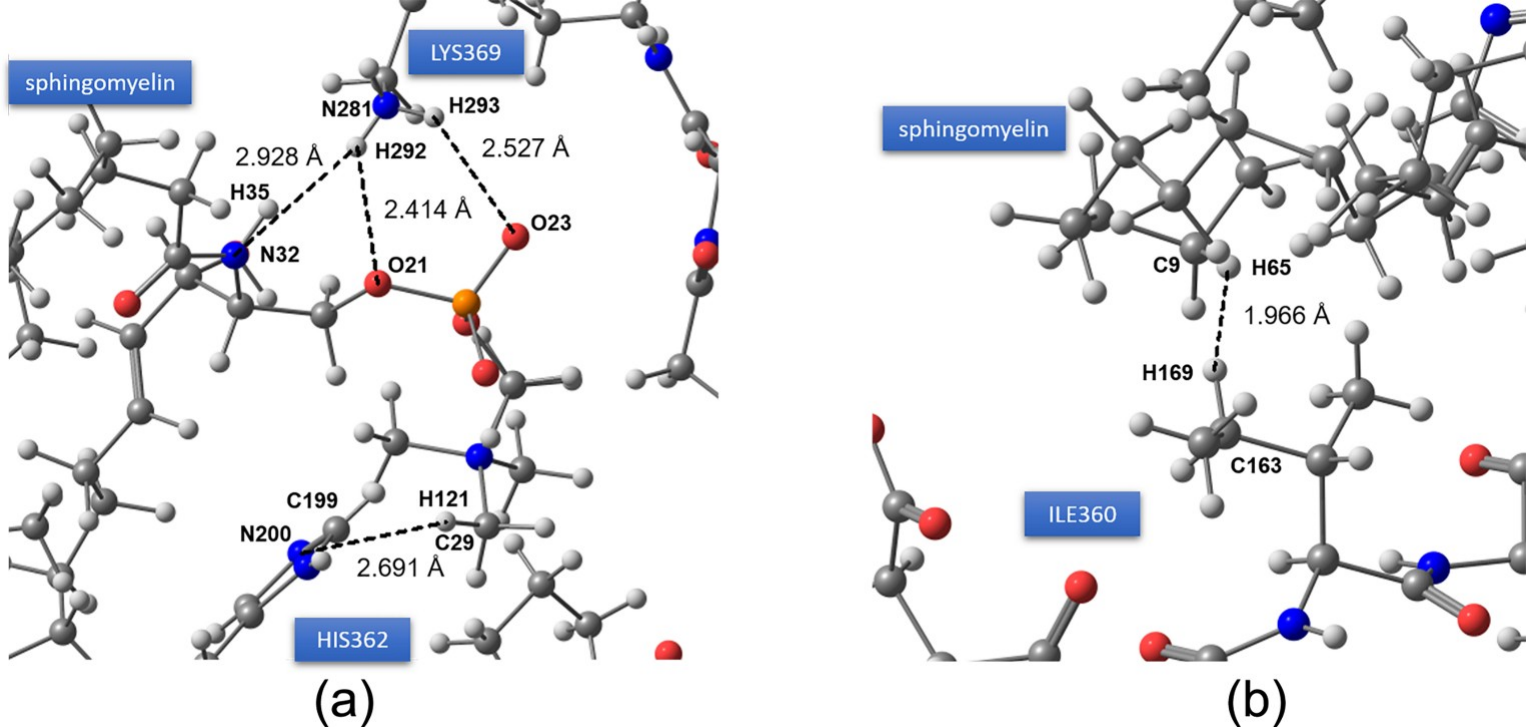
NBO analysis of the intermolecular interactions between PHF-Tau and C18:1 Sphingomyelin. (a) H-bond formation between C18:1 Sphingomyelin and the PHF-Tau backbone and (b) significant London dispersion forces. Interatomic distances are shown in angstrom.

Fig 6A shows the docking of C18:1 Sphingomyelin on PHF-Tau protein. The H-bond formation was from the phosphocholine moiety of C18:1 Sphingomyelin with the protein backbone of the PHF-Tau protofilament, with a binding affinity of -5.4 kcal mol^-1^. The bonding was qualitatively viewed in the protein’s surface map wherein the hydrophilic part of the protein in the middle area with a deep groove forms the binding site S1 (Fig 6B) which was the same site as observed in 1-POPC. Intermolecular interactions based on NBO analysis revealed that the lone pairs of oxygen from the phosphoryl moiety interacted with the amine group of the Lysine having stabilization energies of 1.06 kcal mol^-1^ for (LP(O21) → σ*(N281-H292) and 0.87 kcal mol^-1^ for (LP(O23) → σ*(N281-H293)) (Fig 7A). Another interaction observed is between the amide group of the sphingomyelin and the amine side group of the Lysine (σ(N32-H35) → σ*(N281-H292)) with a stabilization energy of 0.83 kcal mol^-1^. The imidazole moiety of the histidine (HIS362) also contributed a significant intermolecular interaction with an energy of 0.86 kcal mol^-1^ (σ(C199-N200) → σ*(C29-H121)). The stability of the docked conformation was also enhanced by the contribution from London dispersion forces having energies of 0.74 and 0.90 kcal mol^-1^ for interactions σ(C163-H169) → σ*(C9-H65) and σ(C9-H65) → σ*(C163-H169), respectively as shown in Fig 7B.

### 3.2 Molecular dynamics simulation

**Fig 8:**
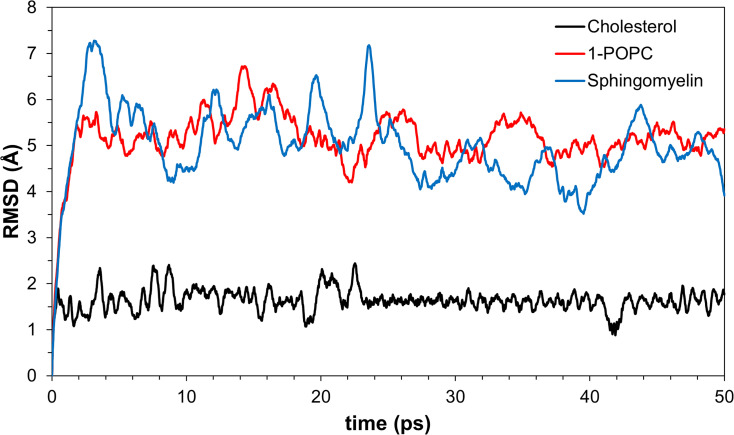
Variations of RMSD values in molecular dynamics simulations of the three membrane lipids.

A molecular dynamics study can provide knowledge of the stability of the protein-ligand complex by analyzing the RMSD of the specified time step. Interestingly, cholesterol has generated lower values of RMSD as compared to the other lipids (Fig 8). The more compact the structure of cholesterol, the less flexible it is as compared to the other lipids. 1-POPC, with structure composed of a phosphocholine moiety and two fatty acid side chains shows to give greater RMSD values upon the start of the simulation which shows its flexibility. Comparable RMSD values of 1-POPC and sphingomyelin docked structures are reached towards the end of the simulation. Based on the simulation.

**Fig 9:**
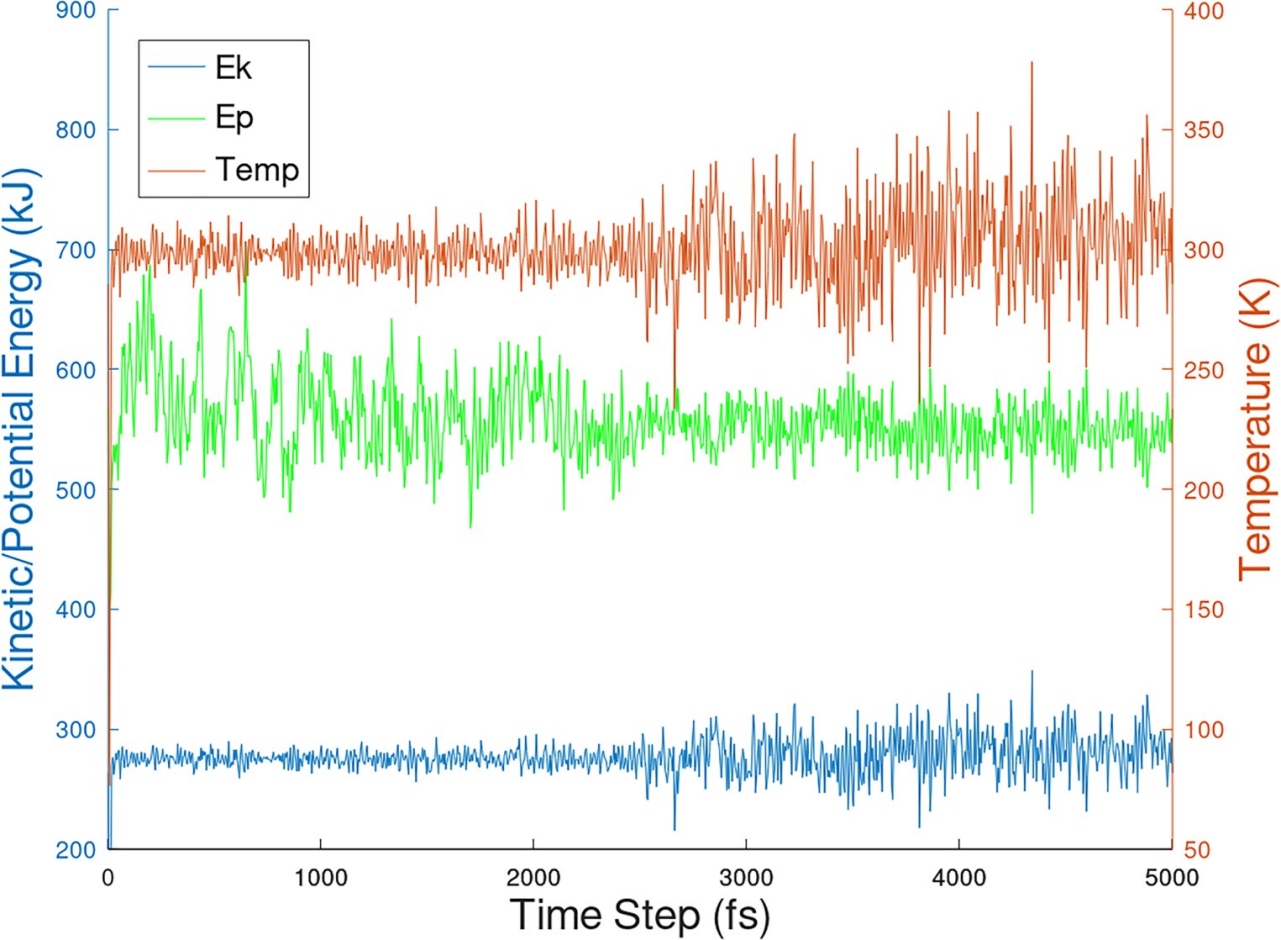
Kinetic, potential and temperature variations in molecular dynamics simulations of of 3-alpha-cholesterol docked structure with RMSD = 0.

**Fig 10:**
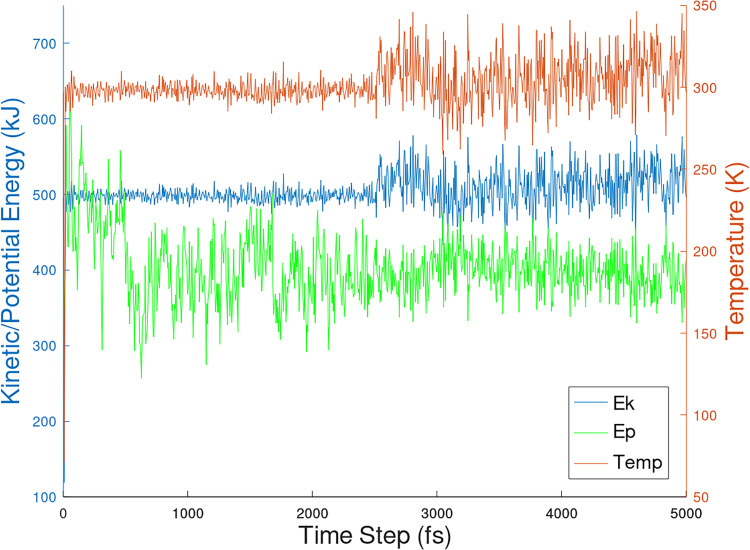
Kinetic, potential and temperature variations in molecular dynamics simulations of 1-POPC docked structure with RMSD = 0.

**Fig 11:**
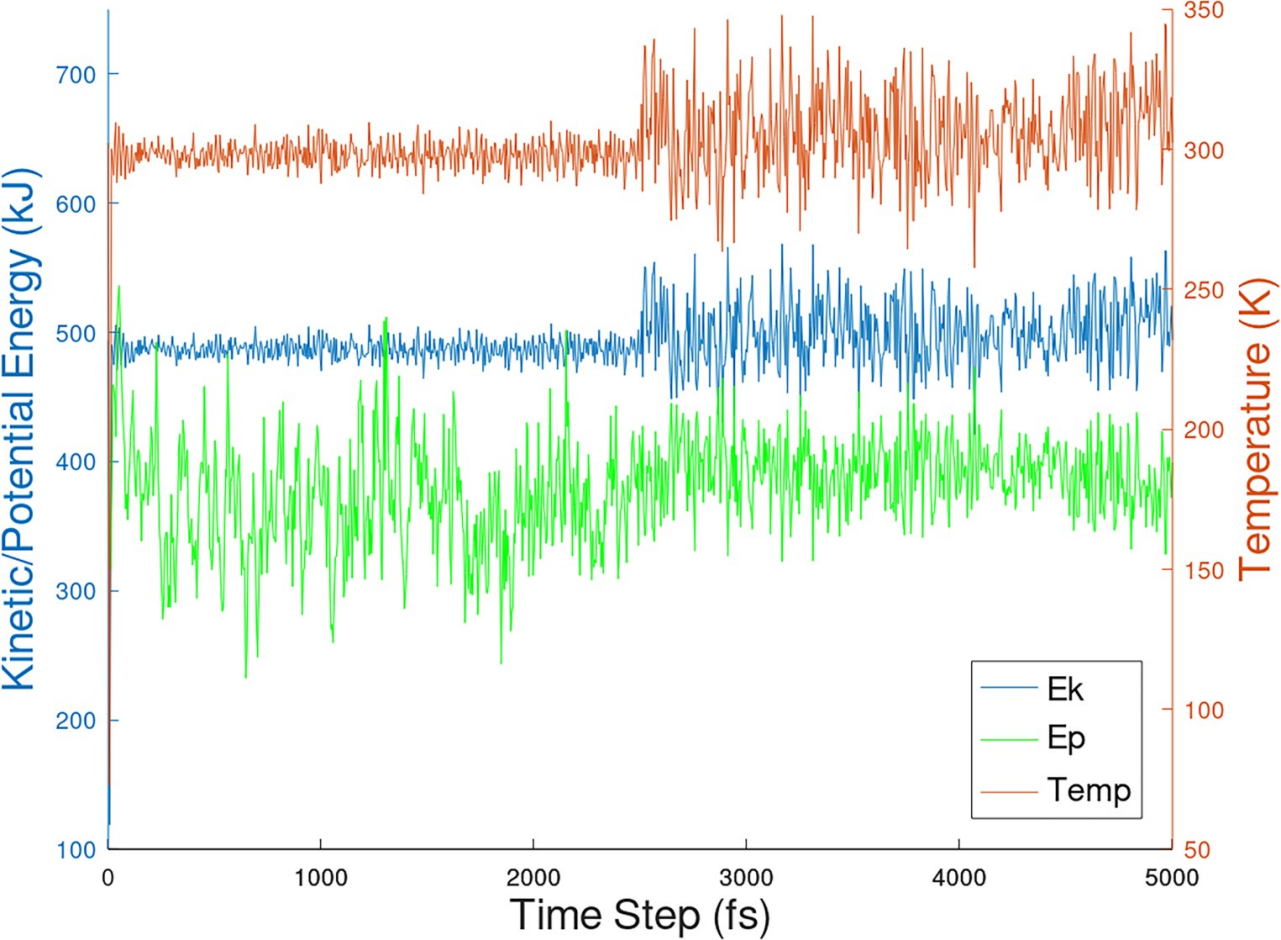
Kinetic, potential and temperature variations in molecular dynamics simulations of C18:1Sphingomelin docked structure with RMSD = 0.

Fig 9 shows the potential and kinetic energies at temperatures starting at 298 K to 300 K obtained at 5 ps time step of the PHF-Tau protofilament/3-alpha-cholesterol complex. Additionally, in Figs 10 and 11, kinetic, potential and temperature variations in molecular dynamics simulations of 1-POPC and C18:1 Sphigomyelin docked structure with RMSD = 0 respectively are shown.

**Fig 12:**
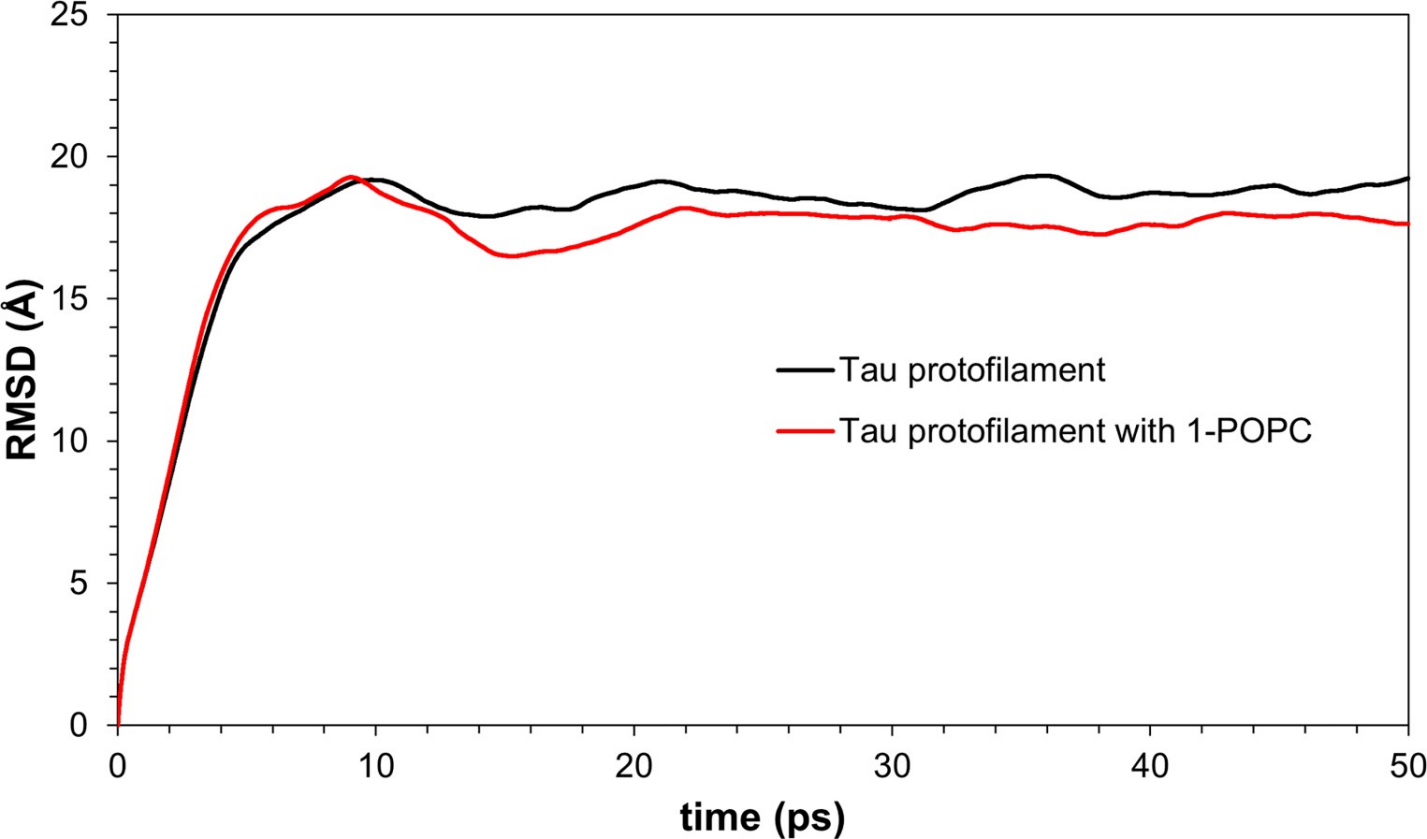
Variations of RMSD values in molecular dynamics simulation of PHF-Tau protofilament and PHF-Tau protofilament with 1-POPC.

The interaction with PHF-Tau to give PHF-Tau/lipid complex generated fairly stable lipid conformations from docking. The MD simulation protocol generates movement of the whole molecule. In Figs 9 to 11, equilibration and production dynamic phase protocols in the MD run can be observed around the middle of the time frame (x-axis) in which the temperature is gradually increased from 298 to 300 K during the equilibration dynamic phase. RMSD values can verify the stability of the molecules during the simulation. The total energy (potential+kinetic) at the given temperature can be seen for cholesterol (Fig 9), 1-POPC (Fig 10) and C18:1 Sphingomyelin (Fig 11) docked structures with the highest binding affinities and lowest RMSD obtained during docking. As temperature increases, the kinetic energy also increases, but the total energy for each lipid is conserved which indicates the stability of the lipids at the binding site. Fig 12 shows the obtained RMSD for the MD simulation of the whole PHF-Tau protofilament with the phospholipid as compared to the RMSD of PHF-Tau protofilament alone. It demonstrates that stable PHF-Tau-phospholipid complex is produced due to lower RMSD values obtained for the complex. The lipid gives additional interactions to the fibril that lowered the flexibility of the fibril. The data is consistent with the reported stable tau(K18)/phospholipid complexes *in vitro* obtained experimentally [34]. K18 is a C-terminal fragment containing the four microtubule-binding repeats of tau [34].

### 3.3 Effect of solvent on docking site

**Fig 13:**
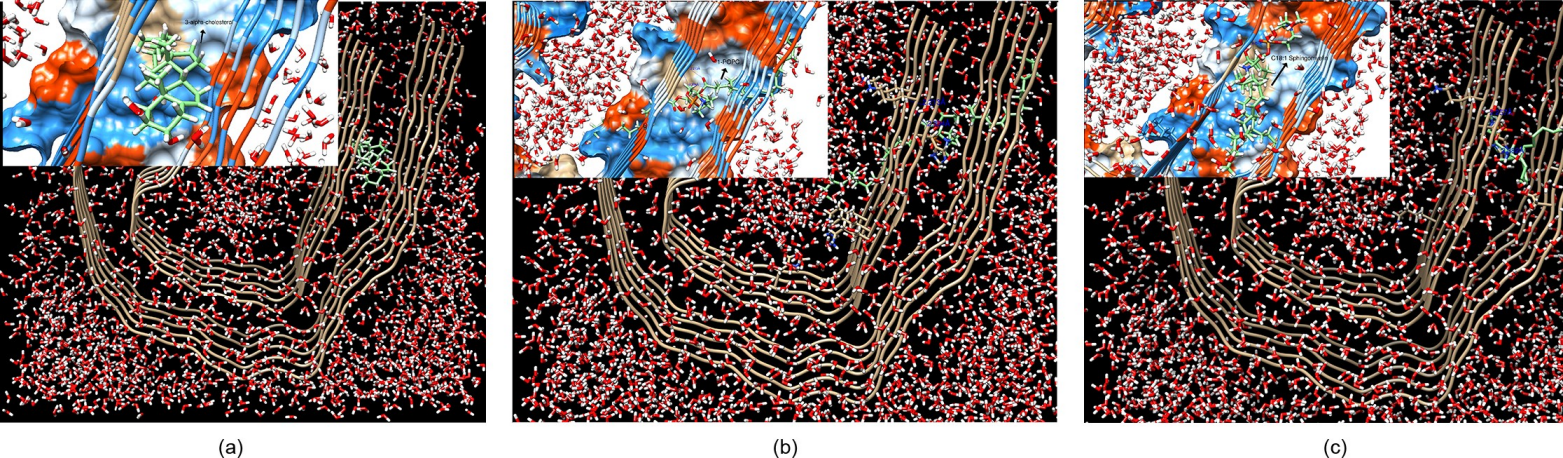
Ligand (AM1-BCC optimized structure) embedded in PHF-Tau protofilament (tail side); RMSD = 0. Hydrophobicity surface is shown. Solvent used is water. Inset shows the preferred configuration of the the ligand: (a) cholesterol; (b) 1-palmitoyl-2-oleoyl-sn-glycero-3-phosphocholine; and (c) C18:1 sphingomyelin and their interactions with the protein.

The tail side of the PHF-Tau protofilament is the side nearest the fuzzy coat that interacts with the extracellular environment [15]. PHF6 (_306_VQIVYK_311_) and the adjacent residues _374_HKLTF_378_ formed from face-face packing of hydrophobic residues [15] and those nearest them are part of the ‘tail side’ (S5 site) (Fig 1). This is the preferred binding site of phospholipid obtained experimentally based on solid-state and solution nuclear magnetic resonance (NMR) spectrometry using the tau(K18)/phospholipid complexes *in vitro* [34]. As shown in Fig 13, the S5 binding site (Fig 1) of PHF-Tau is also the preferred position (RMSD = 0) upon docking the three lipids: cholesterol, sphingomyelin and 1-POPC to the solvated PHF-Tau. The amino acids involved in the H bond interactions obtained here theoretically are VAL313, LYS375, HIS374, ASP314 and TYR310. These residues were also found to interact experimentally, as well as valine (VAL306, VAL309, VAL313, and VAL318), isoleucine (ILE308), and serine (SER316 and SER320) residues [34].

**Fig 14:**
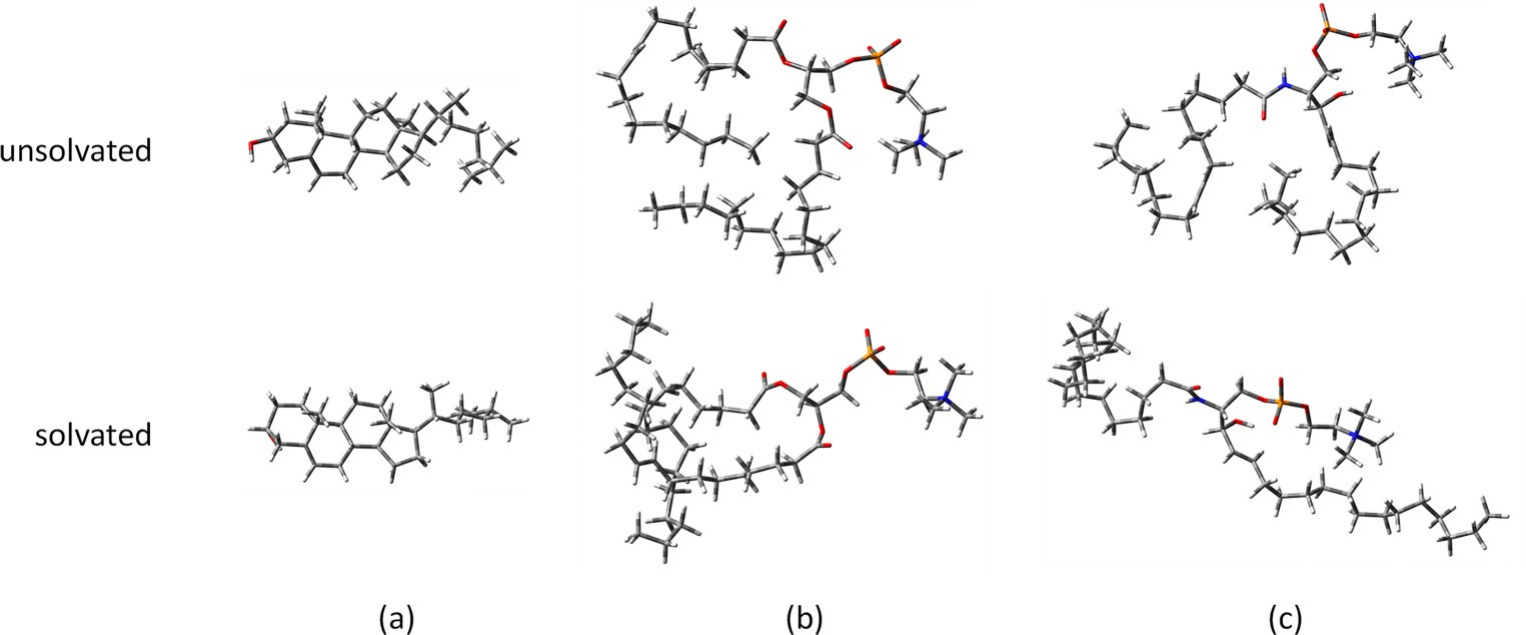
The docked structures of lipids with RMSD = 0 in solvated and unsolvated PHF-Tau. (a) cholesterol; (b) 1-palmitoyl-2-oleoyl-sn-glycero-3-phosphocholine; and (c) C18:1 sphingomyelin.

The differences in the structures of the docked lipids at RMSD = 0 for solvated and unsolvated protofilament can be seen in the Fig 14. Increased binding affinity values are observed upon docking to solvated protein. -10.6 kcal mol^-1^, -5.6 kcal mol^-1^ and -6.1 kcal mol^-1^ are obtained for the binding affinities of 3-alpha-cholesterol, 1-POPC and C18:1 sphingomyelin respectively.

The lipids in PHF-Tau obtained from molecular docking gave different binding affinity values at the different binding sites. For cholesterol, the docked structures are comparable with RMSD = 3.9268 Å (Fig 14A). For the other two lipids (Fig 14B and 14C), the increased RMSD in the unsolvated and solvated docked structures can be attributed to the docking site (embedded at tail site (S5 binding site)) and the effect of solvent. The smaller molecule, cholesterol can be embedded at the tail site without much distortion of its structure.

### 3.4 MD simulation of lipids at tail side

**Fig 15:**
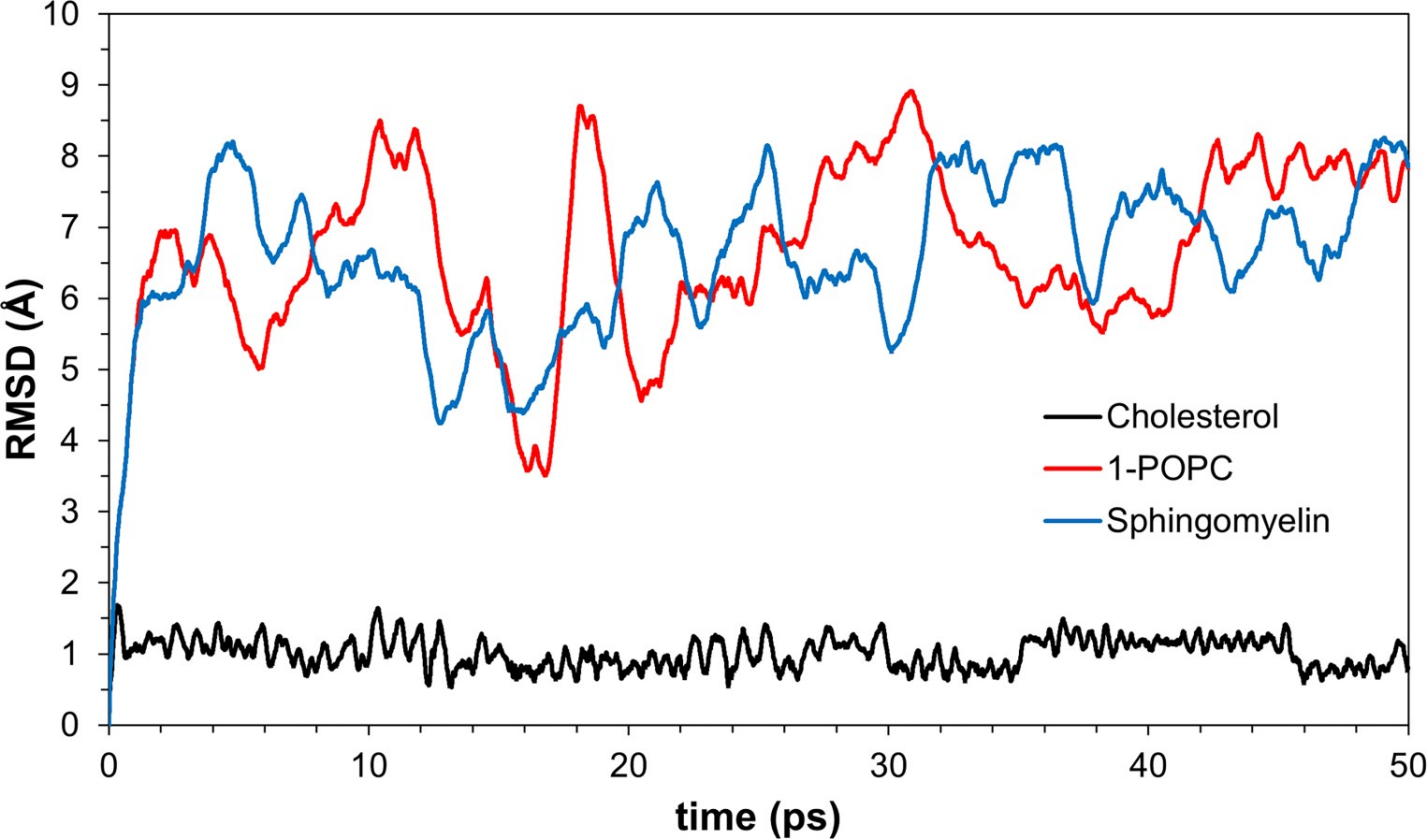
Variations of RMSD values in molecular dynamics simulations of the three membrane lipids in solvated protein.

The docked structures in the solvated protein show comparable RMSD values for cholesterol upon MD simulations to the docked structures in the unsolvated one. The additional water molecules increased the binding affinities of the three lipids to the protein upon docking. The RMSD values of phosphocholine and sphingomyelin upon MD simulations are also increased. The movement of the bigger molecule lipids are affected more than the smaller and more compact one (Fig 15).

## 4. Conclusion

The computationally determined PHF-Tau–lipid complexes, based on the highest binding affinities, NBO analysis, and MD simulations are obtained and are shown to be stable complexes. The docking site of the lipids on unsolvated PHF-Tau protofilament are similar to theoretically obtained binding sites of Tau tracers on unsolvated PHF-Tau. Additional binding site is obtained upon docking to a solvated PHF-Tau, where the it also produced a more stable complexes which are more reflected experimentally. Comparison of the MD simulations of the PHF-Tau protofilament with PHF-Tau protofilament-phospholipid complex indicate a stable tau protein-phospholipid complex is produced. Since the lipids presented in this study are common in normal brain cells, the formation of PHF-Tau protofilament can be harmful. Our assumption is that it is hard to control the aggregation of the Tau protein when it starts. CADD methods such as molecular docking and NBO analysis show to be complementary methods for the system under study. The Tau-aggregation inhibitors can be used to analyze whether they can stop Tau filament aggragation and/or Tau filament-lipid formation. Determinants as to whether or not these molecules disrupt PHF-Tau once it starts to accumulate/aggregate with cell membrane lipids should be established. This study also further supports the use of 1-POPC and Spingomyelin as biomarkers for AD.
